# Identification of Diagnostic Markers for Major Depressive Disorder Using Machine Learning Methods

**DOI:** 10.3389/fnins.2021.645998

**Published:** 2021-06-18

**Authors:** Shu Zhao, Zhiwei Bao, Xinyi Zhao, Mengxiang Xu, Ming D. Li, Zhongli Yang

**Affiliations:** ^1^State Key Laboratory for Diagnosis and Treatment of Infectious Diseases, National Clinical Research Center for Infectious Diseases, Collaborative Innovation Center for Diagnosis and Treatment of Infectious Diseases, The First Affiliated Hospital, Zhejiang University School of Medicine, Hangzhou, China; ^2^Research Center for Air Pollution and Health, Zhejiang University, Hangzhou, China

**Keywords:** biomarkers, depression, machine learning, major depressive disorder, meta-analysis

## Abstract

**Background:**

Major depressive disorder (MDD) is a global health challenge that impacts the quality of patients’ lives severely. The disorder can manifest in many forms with different combinations of symptoms, which makes its clinical diagnosis difficult. Robust biomarkers are greatly needed to improve diagnosis and to understand the etiology of the disease. The main purpose of this study was to create a predictive model for MDD diagnosis based on peripheral blood transcriptomes.

**Materials and Methods:**

We collected nine RNA expression datasets for MDD patients and healthy samples from the Gene Expression Omnibus database. After a series of quality control and heterogeneity tests, 302 samples from six studies were deemed suitable for the study. R package “MetaOmics” was applied for systematic meta-analysis of genome-wide expression data. Receiver operating characteristic (ROC) curve analysis was used to evaluate the diagnostic effectiveness of individual genes. To obtain a better diagnostic model, we also adopted the support vector machine (SVM), random forest (RF), k-nearest neighbors (kNN), and naive Bayesian (NB) tools for modeling, with the RF method being used for feature selection.

**Results:**

Our analysis revealed six differentially expressed genes (*AKR1C3*, *ARG1*, *KLRB1*, *MAFG*, *TPST1*, and *WWC3*) with a false discovery rate (FDR) < 0.05 between MDD patients and control subjects. We then evaluated the diagnostic ability of these genes individually. With single gene prediction, we achieved a corresponding area under the curve (AUC) value of 0.63 ± 0.04, 0.67 ± 0.07, 0.70 ± 0.11, 0.64 ± 0.08, 0.68 ± 0.07, and 0.62 ± 0.09, respectively, for these genes. Next, we constructed the classifiers of SVM, RF, kNN, and NB with an AUC of 0.84 ± 0.09, 0.81 ± 0.10, 0.73 ± 0.11, and 0.83 ± 0.09, respectively, in validation datasets, suggesting that the SVM classifier might be superior for constructing an MDD diagnostic model. The final SVM classifier including 70 feature genes was capable of distinguishing MDD samples from healthy controls and yielded an AUC of 0.78 in an independent dataset.

**Conclusion:**

This study provides new insights into potential biomarkers through meta-analysis of GEO data. Constructing different machine learning models based on these biomarkers could be a valuable approach for diagnosing MDD in clinical practice.

## Introduction

From 1990 to 2016, major depressive disorder (MDD) was one of the five leading causes of years lived with disability (GBD 2016 Disease and Injury Incidence and Prevalence Collaborators, 2017). Patients with MDD have a higher risk of diabetes, stroke, cardiovascular disease, obesity, cancer, cognitive impairment, and Alzheimer’s disease (Otte et al., 2016). Moreover, MDD is one of the most common disorders associated with suicidal behavior. It has been estimated that the risk of suicide in MDD patients is increased substantially (greater than 10 times) compared with the general population (Chesney et al., 2014).

Early diagnosis and appropriate treatment would undoubtedly reduce the incidence and mortality rate of MDD patients. However, like many other affective disorders, the complex etiology of MDD and the inevitable need for clinical judgment based on an individual’s medical history may cause a lack of reliability in diagnosis. More objective diagnostic methods thus are required. Previous studies have explored molecular biomarkers of MDD based on genomic, epigenetic, transcriptomic, and proteomic sources (Gururajan et al., 2016). Several types of molecules have been revealed with these approaches, which include mitochondrial DNA (Cai et al., 2015), small non-coding RNAs (Bocchio-Chiavetto et al., 2013), neurotransmitters (Belzeaux et al., 2010), neurotrophic and growth factors (Iga et al., 2007; Cattaneo et al., 2013), HPA axis-related molecules (Austin et al., 2003), and mediators of neuroinflammation (Cattaneo et al., 2013; Iacob et al., 2013). For example, several studies (Cattaneo et al., 2013; Iacob et al., 2013, 2014) reported increased expression of peripheral mRNAs for the pro-inflammatory cytokines interleukin (IL)-1α, IL-1β, IL-6, IL-8, IL-10, interferon (IFN)-γ, migration inhibitory factor (MIF), and tumor necrosis factor (TNF)-α in MDD patients compared with healthy control subjects. In addition, neuroimaging approaches, such as magnetic resonance imaging (MRI), electroencephalography (EEG), diffusion tensor imaging (DTI), near-infrared spectroscopy (NIRS), and molecular imaging (i.e., PET and SPECT) (Kang and Cho, 2020) have been used to discover biomarkers for diagnosis and treatment of MDD.

This study focused on the peripheral transcriptomic biomarkers, which have been described as “sentinels of disease” (Liew et al., 2006). Because of the complicated and heterogeneous pathogenesis of MDD, there existed some limitations in the study of relevant transcriptomic biomarkers. For example, studies on brain-derived neurotrophic factor (*BDNF*) had inconsistent results. The studies of Karege et al. (2005) and Piccinni et al. (2008) reported a reduction in BDNF in depressed patients compared with healthy persons, but in the study of Serra-Millàs et al. (2011), MDD patients showed higher plasma BDNF concentrations. However, in another study, researchers found no significant difference in plasma BDNF concentrations between MDD patients and control subjects (Bocchio-Chiavetto et al., 2010). Based on these facts, it appears that identification of reliable biomarkers for predicting diagnosis and treatment of MDD remains a challenge.

Therefore, in this study, meta-analysis was first performed to identify consistent biomarkers from different large-sample datasets. Although there are various meta-analyses of microarray data, they generally focus on one or a few genes; few have been developed for systematic integration of multiple microarray datasets (Sun et al., 2017; Li et al., 2018). The current commonly used meta-analysis method was proposed by Choi et al. (2003) and facilitates the detection of small but consistent expression changes and increases sensitivity and reliability. With this method, Chen et al. (2019) acquired gene expression data from eight commonly used *in vitro* macrophage models to perform a meta-analysis and identified consistently differentially expressed genes (DEGs) that have been implicated in inflammatory and metabolic processes. Forero et al. (2017) found MDD-related DEGs for blood, amygdala, cerebellum, anterior cingulate cortex, and prefrontal cortex regions based on GWES using meta-analysis. However, whether these DEGs would be useful as biomarkers has not been evaluated yet.

MDD is influenced by both genetics and environment where the transcriptome feature patterns or feature function patterns may represent disease subtypes, outcome prognosis, drug benefit prediction, or specific biological process. The machine learning (Anttila et al., 2018) approach has an advantage in recognizing subtle patterns in large and noisy datasets, which is particularly useful in the study of complex transcriptome data. For example, Xu et al. (2012) employed the SVM-RFE approach to select genes for prediction of breast cancer prognosis and discovered a 50-gene signature that yielded significantly higher accuracy than the widely used 70-gene signature (van ‘t Veer et al., 2002). By adopting fuzzy forests of transcriptome data, Ciobanu et al. (2020) found that the downregulated *TFRC* (transferrin receptor) can predict recurrent MDD with an accuracy of 63%.

The aim of this study was to identify potential transcriptional biosignatures that might be used for the diagnosis of MDD. Here, we applied meta-analysis to discover DEGs differing between MDD patients and healthy controls. Six significant DEGs with FDRs < 0.05 were investigated for their diagnostic capability. To obtain better diagnostic efficacy, we compared four ML models. Finally, an SVM prediction model consisting of 70 feature genes was constructed and validated by a reserved independent gene expression dataset.

## Materials and Methods

### Systematic Search of Microarray Expression Profiling Datasets

**FIGURE 1:**
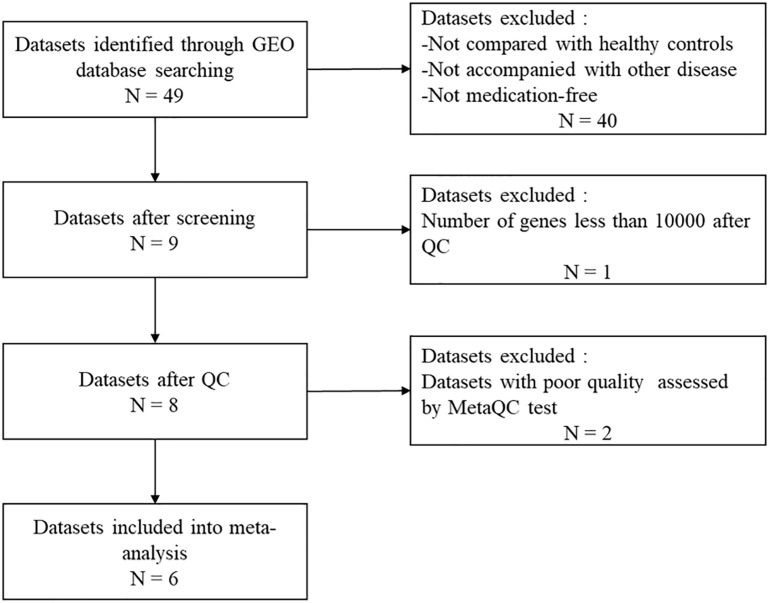
Workflow of data processing. GEO, Gene Expression Omnibus; QC, quality control.

MDD-related keywords were searched in the Medical Subject Headings(MeSH) library^1^. Then we conducted a systematic search in the GEO repository^2^ using the following search sentence: ((((((((MDD) OR major depressive disorders) OR depressive disorders) OR depressive syndromes) OR depression)) AND (((blood) OR peripheral blood) OR PB)) AND Homo sapiens [Organism]) AND Expression profiling by array [Filter]. A report was included in the analysis if the following criteria were satisfied: (1) used a case-control design; (2) patients did not have any diseases other than MDD; and (3) the patients were medication free. We finally obtained a total of 9 datasets (Figure 1).

### Initial Data Processing

Nine microarray datasets were retrieved from the GEO database: GSE98793, GSE19738, GSE38206, GSE52790, GSE39653, GSE76826, GSE58430, GSE32280, and GSE46743, with a sample size of 128, 67, 18, 22, 45, 22, 12, 16, and 160, respectively (Table 1).

**TABLE 1:** Basic information of collected microarray datasets.

Study	GEO accession number	Country	Array platform	Samples MDD/Control	Number of genes after QC
Leday et al. (2018)	GSE98793	United Kingdom	Affymetrix Human Genome U133 Plus 2.0 Array	64/64	20188
Spijker et al. (2010)	GSE19738	Netherlands	Agilent-012391 Whole Human Genome Oligo Microarray G4112A	33/34	13334
Belzeaux et al. (2012)	GSE38206	France	Agilent-028004 SurePrint G3 Human GE 8x60K Microarray	9/9	33074
Liu et al. (2014)	GSE52790	China	Affymetrix Human hGlue_3_0_v1 Array	10/12	16951
Savitz et al. (2013)	GSE39653	United States	Illumina HumanHT-12 V4.0 expression beadchip	21/24	29328
Miyata et al. (2016)	GSE76826	Japan	Agilent-039494 SurePrint G3 Human GE v2 8x60K Microarray 039381	10/12	27382
Arloth et al. (2015)	GSE46743	Germany	Illumina HumanHT-12 V3.0 expression beadchip	69/91	8615
Wang et al. (2015)	GSE58430	China	Agilent-028004 SurePrint G3 Human GE 8x60K Microarray	6/6	20188
Yi et al. (2012)	GSE32280	China	Affymetrix Human Genome U133 Plus 2.0 Array	8/8	22879

For the GSE98793, GSE52790, and GSE32280 datasets, based on the Affymetrix platform (Thermo Fisher Scientific, Inc., Waltham, MA, United States), the raw CEL data were downloaded; and the Robust Multi-Array Average (RMA) method and the ‘‘Oligo’’ package from BioConductor^3^ were used to normalize the data and annotate the probe information. For the GSE19738, GSE38206, GSE58430, and GSE76826 data, based on the Agilent platform (Agilent Technologies, Inc., Santa Clara, CA, United States), the quantile method was used to normalize the data. Annotation of the probe information was based on Agilent platform information. For the GSE39653 and GSE46743 datasets based on the Illumina platform (Illumina Inc., San Diego, CA, United States), the quantile method was used to normalize the data, and annotation of the probe information was based on Illumina platform information.

Normalized signal intensity data were imported into BRB-Array Tools (v. 4.5)^4^ for initial processing. We excluded those genes with more than 50% of the data missing. The most variable probe measured by inter-quartile range (IQR) was used to handle redundant probe sets that correspond to the same gene.

### Microarray Gene Expression Meta-Analysis

Meta-analysis of microarray data was carried out in “MetaOmics” based on R language (Ma et al., 2019), which includes three packages: MetaQC, MetaDE, and MetaPath. MetaQC (Kang et al., 2012) was used for the quality control of datasets before meta-analysis, and the MetaDE (Wang et al., 2012) package was used to identifying differentially expressed genes.

We used the following six quantitative quality control indexes to assess heterogeneity across different studies: internal homogeneity of co-expression structure among studies (IQC), external consistency of co-expression pattern with pathway database (EQC), accuracy of biomarker detection (AQCg), accuracy of enriched pathway detection (AQCp), consistency of differentially expressed genes (CQCg), and consistency of enriched pathway ranking (CQCp). Each QC index was defined as the minus log-transformed *p*-value from formal hypothesis testing in each QC criterion (Wang et al., 2012). Finally, standardized mean rank (SMR) was generated to assist decision making. In this study, datasets with SMR values > 5 were excluded from analysis (Esmaeili et al., 2020).

MetaDE was used for identifying differentially expressed genes, and the meta-analysis method used in the current study was developed by Choi et al. (2003). The change of gene expression was represented as “effect size,” a standardized index measuring the magnitude of a treatment or covariate effect. The effect sizes of different studies were combined to obtain an estimate of the overall mean. Herein, we applied the random effects model, and FDR correction was used to control for multiple testing. Finally, genes were considered significant at FDR < 0.05.

### Protein–Protein Interaction (PPI) Network Construction and Module Analysis

A total of 217,249 pairs of FIs were downloaded from Reactome (v. 2014^5^; Croft et al., 2011). These pairwise relations were derived from datasets of protein–protein interactions in BioGrid (Chatr-Aryamontri et al., 2015), the Database of Interacting Proteins (Salwinski et al., 2004), the Human Protein Reference Database (Keshava Prasad et al., 2009), I2D (Brown and Jurisica, 2007), IntACT (Orchard et al., 2014), and MINT (Licata et al., 2012), as well as from gene co-expression data derived from multiple high-throughput techniques, including yeast two-hybrid assays, mass spectrometry pull-down experiments, and DNA microarrays (Wu et al., 2010). The above interaction information was imported into Cytoscape software (v. 3.2.1^6^) to construct the FI network (Shannon et al., 2003). A spectral partition-based network clustering (Newman, 2006) was used to search for modules based on the FI network. A KEGG pathway enrichment analysis was used to analyze functions for each individual network module. We selected a size cutoff of 2 to filter out small network modules. An FDR value of < 0.05 was considered to represent significantly enriched processes or signaling pathways. Co-expression patterns of the genes in the same module were analyzed by using the Pearson correlation test.

### Establishment of the ML Classifier

Supplementary Figure 3 shows an overview of the proposed ML method involving feature extraction, selection, classification, and validation. R package ‘‘caret’’ (v. 6.0-84^7^) was applied in the following steps.

The six datasets included in this study were divided into two parts: GSE98793 (Leday et al., 2018), GSE19738 (Spijker et al., 2010), GSE39653 (Savitz et al., 2013), GSE52790 (Liu et al., 2014), and GSE76826 (Miyata et al., 2016) were used as discovery datasets and GSE38206 (Belzeaux et al., 2012) as an independent validation dataset. We performed meta-analysis on discovery sets to identify DGEs between MDD patients and healthy controls. DEGs with *p* < 0.01 were considered potential biomarkers for further feature selection. Then, the dataset GSE98793 with the largest sample size was used as the training dataset in both feature screening and ML modeling. The other four datasets in discovery sets were used for internal validation.

We applied an RF algorithm to reduce the number of feature genes, with the following steps: (1) ranked genes in descending order based on their importance; (2) eliminated feature genes one by one according to the importance of each feature with the goal of producing a new feature set; and (3) repeated the above process with the new feature set. We used 10-fold cross-validation for verification and calculated the average accuracy value to assess the classification capability. Finally, the feature set with the highest average accuracy was selected for model construction.

The following ML algorithms, SVM (Cortes and Vapnik, 1995), RF (Tin Kam, 1998), kNN (Altman, 1992), and NB (Friedman et al., 1997), were used to build prediction models for gene expression data.

The aim of SVM algorithm is to identify a decision hyperplane that make the distance between the hyperplane and the instances that are closest to boundary is maximized. By introducing the concept of “soft margin” and using “kernel trick,” SVM performs well with linear indivisibility data (Boser et al., 1996). SVM with the radial basis function (RBF) kernel was used in this study and parameters “C” and “sigma” were returned.

Random Forest is an ensembles-learning algorithm forming with a series of decision trees. Each tree is developed from a subset from the training data. The class of the new instance is determined by using the majority vote of individual trees in the forest (Tin Kam, 1998). In this study, R package “randomForest” was used in the modeling and we tuned the parameter “mtry.”

In kNN, classification is based on the distance between the instances and an object is classified according to the status of its k nearest neighbors. R package “kknn” was used in the modeling, where we set distance = 2 (Euclidean Distance), and tuned the parameter “kmax.”

Naive Bayes is a statistical classification algorithm based on theBayes theorem. The core idea of Naive Bayes is, if the probability ofinstance x belonging to A is greater than the probability of belonging to B under some attribute conditions, it is said that instance x belongs to A. R package “klaR” was used in the modeling and output parameter “usekernel.”

Default values were used for other parameters in each model. To evaluate the overall performance of each model, leave-one-out cross-validation was performed. Details about the tested parameters and their corresponding test values for each model are provided in Supplementary Table 6.

We used the average AUC to assess the classification capability of each model. Finally, a model with the highest average AUC in validation sets was chosen.

To facilitate clinical application, we attempted to construct a model with fewer genes without affecting the accuracy of classification efficiency. We compared the classification ability of the model based on the average AUC value of discovery set. The criterion for determining the final model was that the model achieved the optimal average AUC value in the discovery sets.

The feature genes in the final determined SVM classifier were used to perform the supervised clustering of samples and extents of expression. The clustering results were visualized using a heatmap (Gu et al., 2016).

## Results

### Data Sets Collection and Pre-processing

The information in the datasets is shown inTable 1. Dataset GSE46743 (Miyata et al., 2016) has a number of genes < 10,000 after QC was excluded. Eight eligible datasets were finally used for the following analysis, which consisted of a total of 161 MDD and 169 control samples.

### Microarray Gene Expression Meta-Analysis

**FIGURE 2:**
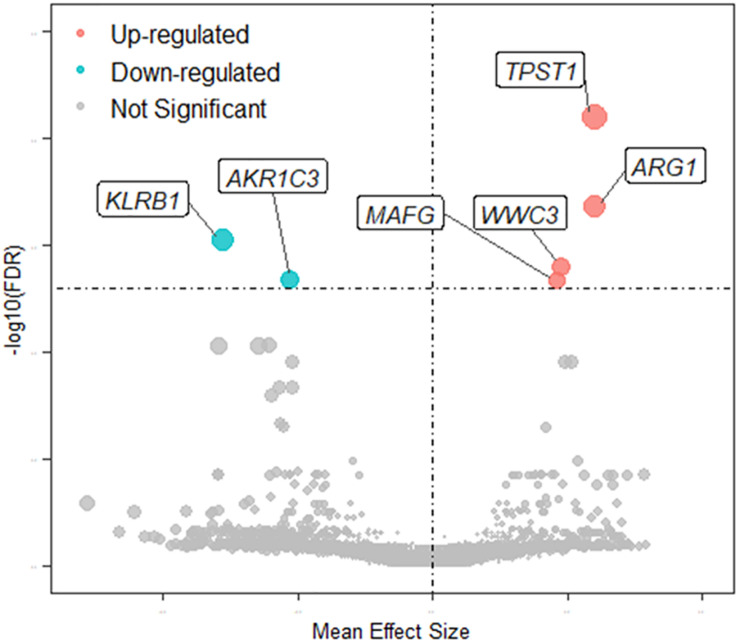
Volcano plot of MDD-related DEGs. Node colors define change direction in DEGs: red for upregulated genes, green for downregulated genes, and gray for not significant genes. Node size combines the effect size and FDR value: a larger node indicates that mean effect size of gene is large and FDR value is small.

**FIGURE 3:**
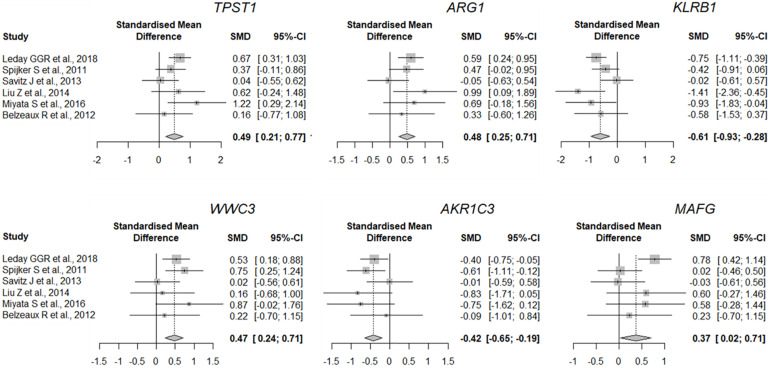
Expression of most significant DEGs between MDD and control group (a random-effects model) in different studies. Lines indicate 95% confidence intervals (CI), and the midpoint of each line is denoted by a square indicating the standardized mean difference (SMD) for each study. Diamond indicates overall SMD and 95% confidence interval.

The quality of the eight datasets was assessed utilizing “MetaQC.” Among the eight microarray datasets, six were included in the further meta-analysis for DEGs (Supplementary Figure 1 and Supplementary Table 1), and the other two studies (Yi et al., 2012; Wang et al., 2015) were excluded because of their lower quantitative quality control scores (SMR < 5). We then combined the other six datasets and obtained a total of 9,263 common genes that were used as input for the meta-analysis, which revealed 137 DEGs with *p* < 0.01. Of them, 66 were upregulated and 71 downregulated in MDD (Figure 2). A detailed list of these DEGs is given in Supplementary Table 2. Figure 2 shows the six most significant DEGs with FDRs < 0.05; they are tyrosylprotein sulfotransferase 1 (*TPST1*), arginase 1 (*ARG1*), killer cell lectin-like receptor B1 (*KLRB1*), WWC family member 3 (*WWC3*), aldo-keto reductase family 1 member C3 (*AKR1C3*), and MAF bZIP transcription factor G (*MAFG*). The forest plots of the six genes’ expression in different datasets are shown in Figure 3. These six genes were associated with immune process, inflammatory response, and hormonal metabolic process (Supplementary Table 3). In short, these results implied that these six biomarkers might play important roles in MDD.

### Protein–Protein Interaction Network and Module Pathway Enrichment

By mapping 137 MDD-related DEGs to the FI data, we constructed an MDD-related FI network comprising 137 nodes, of which 103 were isolated and 34 were classified into seven clusters (Supplementary Figure 2A). A topographical analysis of the FI network revealed five modules ranging in size from three to eight genes (Supplementary Figure 2B). We next explored the potential co-expression of the DEGs in each module. There was a moderate to high positive correlation among the expression of most genes in Modules 0, 3, and 4 (Supplementary Figure 2C). In Module 1, there was a moderate to high positive correlation among *MLKL*, *CEP63*, and *CSNK1E*, a moderate negative correlation among *DNAJC7*, *MLKL*, and *CSNK1E* (Supplementary Figure 2C). Most genes in Module 2 showed a low correlation between each other. To understand how the 26 genes of the five modules were related to the molecular mechanisms of MDD, we performed a functional enrichment analysis of these modules based on pathway annotation (Supplementary Table 4). The enriched pathways of Modules 0 and 2 were related to some elements and events in transcription and translation, such as the ribosome, spliceosome, RNA degradation, or mRNA surveillance pathway. Similarly, genes in Module 3 were involved in transcriptional mis-regulation in cancers. Module 1 was related mainly to signaling pathways associated with the immune response, for example, antigen processing and presentation and the IL-17 signaling pathway. Neurodegenerative disease-related pathways in KEGG were enriched in Module 4, which included genes involved in Alzheimer, Huntington, and Parkinson diseases. In addition, metabolic pathways were enriched in Module 4. Together, this identified MDD-related FI network of dysregulated pathways could serve as a pool of novel functional module genes for future investigation in the diagnosis of MDD.

### Evaluation of Diagnostic Ability of Single Genes

Next, we constructed single gene models to distinguish MDD patientsfrom healthy control subjects. We chose the six most significant DEGsand calculated the diagnostic ability of these genes with ROC curveanalysis. Table 2 shows the predicted resultsof single gene model in different datasets, represented by AUC values. The gene with the most significant expression difference, *TPST1*, had an average AUC value of 0.68 and a predictive ability of 0.82 in the dataset of Miyata et al. (2016), but only 0.62 in the dataset of Belzeaux et al. (2012). The gene with the better predictive potency was *KLRB1*, with an average AUC value of 0.70 and an SD of 0.11. The performance of *WWC3* was the worst, with an average AUC of 0.63 and an SD of 0.04. These results indicated that the model developed with individual genes was not effective for diagnosis of MDD in clinics.

**TABLE 2:** AUC of single gene models for MDD diagnosis.

Study	*TPST1*	*ARG1*	*KLBR1*	*WWC3*	*AKR1C3*	*MAFG*
Leday et al. (2018)	0.67	0.66	0.70	0.65	0.60	0.71
Spijker et al. (2010)	0.63	0.62	0.59	0.71	0.66	0.50
Savitz et al. (2013)	0.65	0.57	0.55	0.51	0.65	0.61
Liu et al. (2014)	0.68	0.72	0.85	0.51	0.67	0.68
Miyata et al. (2016)	0.82	0.68	0.72	0.72	0.63	0.62
Belzeaux et al. (2012)	0.62	0.78	0.78	0.62	0.56	0.69
Mean ± SD	0.68 ± 0.07	0.67 ± 0.07	0.70 ± 0.11	0.62 ± 0.09	0.63 ± 0.04	0.64 ± 0.08

### ML Classifier

The above analyses indicated that the average AUCs of most single gene models were less than 0.70, suggesting that more efficient diagnostic models were necessary. We used the transcriptome data from the discovery sets for meta-analysis and obtained 114 DEGs (Supplementary Table 5) with *p* < 0.01 as input for feature screening. First, the feature set containing 108 genes was selected as it had the highest accuracy in the training dataset (Supplementary Figure 4A). Based on this feature set, four ML classification methods were used for modeling and the parameters of each model were shown in Supplementary Table 6. All models yielded an average AUC > 0.7 in validation datasets (Table 3), with SVM producing the highest average AUC (0.84). We thus chose SVM as the final diagnostic model.

**TABLE 3:** Comparison of different models in the validation sets.

Average value	SVM	kNN	NB	RF
AUC	0.84 ± 0.09	0.73 ± 0.11	0.83 ± 0.09	0.81 ± 0.10
Accuracy	0.79 ± 0.11	0.69 ± 0.10	0.74 ± 0.13	0.76 ± 0.12
Sensitivity	0.80 ± 0.14	0.54 ± 0.16	0.81 ± 0.15	0.83 ± 0.14
Specificity	0.77 ± 0.10	0.82 ± 0.07	0.69 ± 0.14	0.70 ± 0.14

**FIGURE 4:**
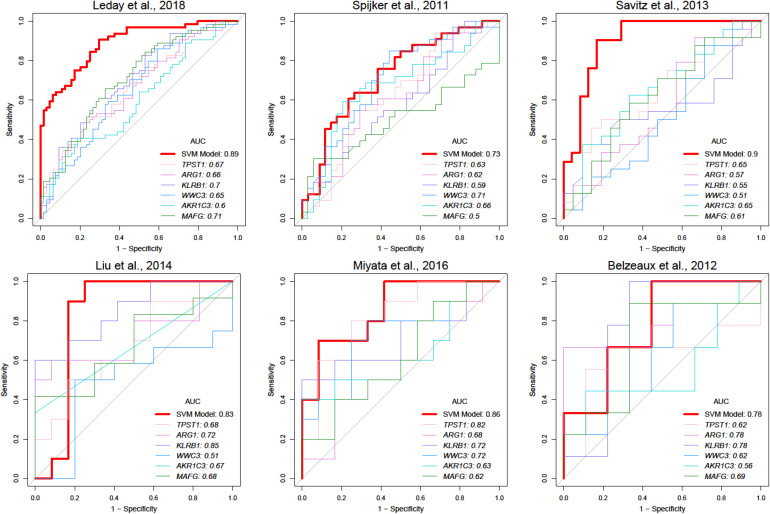
Comparison of prediction performance between SVM and single-gene models. Red lines represent ROC curves of SVM model in different studies, and lines with other colors represent ROC curves of various single gene models.

To facilitate clinical application, we attempted to construct a modelwith fewer genes. Feature genes ranked with an average AUC ofdiscovery datasets were picked at 10 intervals from the top 10 to number 108. As shown in Supplementary Figure 4B, the accuracy of the SVM classifier was improved with an increasing number of genes, and the average accuracy reached the top at 0.84 once 70 genes were selected. The SVM classifier was still able to distinguish MDD samples from the healthy controls in test datasets with an average AUC of 0.82, accuracy of 0.75, sensitivity of 0.78, and specificity of 0.74 (Table 4). In the independent dataset, the classifier achieved an AUC of 0.78 (Table 4), which was greatly better than the model with randomly selected 70 genes (Supplementary Table 9). Besides, we calculated positive predictive value (0.74 ± 0.06) and Matthews correlation coefficient (0.52 ± 0.14) in training and test data sets which also reflect a great performance of our model (Supplementary Table 8). The top six most significant genes mentioned above were all included in this SVM model (Supplementary Figure 5 and Supplementary Table 7). Compared with a single gene, the SVM model had better predictive performance (Figure 4).

**TABLE 4:** Evaluation of classification effect of the SVM model.

Testing sample	Study	AUC	Accuracy	Sensitivity	Specificity
Training	Leday et al., 2018	0.89	0.77	0.78	0.77
Internal test	Spijker et al., 2010	0.73	0.67	0.73	0.62
	Savitz et al., 2013	0.91	0.80	0.90	0.71
	Liu et al., 2014	0.83	0.86	0.90	0.83
	Miyata et al., 2016	0.86	0.77	0.70	0.83
Independent test	Belzeaux et al., 2012	0.78	0.67	0.67	0.67
Mean ± SD in test datasets		0.82 ± 0.07	0.75 ± 0.08	0.78 ± 0.11	0.74 ± 0.10

## Discussion

Although there have been numerous reports analyzing DEGs in MDD patients and healthy individuals, an exploration of diagnosis and etiology of MDD remains a challenge. To identify effective diagnostic biomarkers of MDD, in this study, we first conducted a meta-analysis of six studies, which revealed 137 DEGs with a *p* < 0.01. Then we identified functional module genes showing that these DEGs were involved in the processes of transcription and translation, inflammation, immune-related pathways, and neurodegenerative diseases. Six DEGs with FDR < 0.05 were investigated by ROC curve analysis for their potential to distinguish MDD patients from healthy controls. To improve predictive power, we applied four ML methods with RF being used for feature selection. Finally, we constructed an SVM model containing 70 feature genes and showed that it was superior to the single gene prediction model.

In the first part of the present study, we used meta-analysis to identify reliable DEGs as biomarkers. Six differentially expressed genes with FDRs < 0.05 were identified in MDD and healthy control subjects and used to calculate classification efficiency. Among them, the functions of *KLRB1*, *ARG1*, and *TPST1* were associated with immune and inflammatory responses (Supplementary Table 2). It is known that inflammation and immunity play an important role in the etiology of depression (Liu et al., 2019). For example, individuals with autoimmune diseases and severe infections are more likely to have depression (Malhi and Mann, 2018). Peripheral cytokine concentrations have been linked to brain function, wellbeing, and cognition (Bollen et al., 2017). Therefore, we speculate that *KLRB1*, *ARG1*, and *TPST1* influence the occurrence and progression of MDD by participating in the immune or inflammation pathways. *AKR1C3* has the activity of aldo-keto reductase (nicotinamide adenine nucleotide phosphate; NADP), which plays an important role in interconversion of androgens, estrogens, and progestins to their cognate inactive metabolites (Supplementary Table 2). Over the past several years, both clinical and preclinical studies have established a strong link between sex hormones and depression (Thériault and Perreault, 2019). For example, in women, the prevalence of depression correlates with changes in hormonal fluctuations, such as puberty, prior to menstruation, during the postpartum period, and after the onset of menopause (Thériault and Perreault, 2019). Even if these differentially expressed genes are biologically related to the MDD process, ROC analysis showed that the diagnostic AUC value of a single gene was generally < 0.7, and, importantly, there existed great differences in the effectiveness of a diagnostic model developed on the basis of individual genes among different datasets or studies. These results strongly indicated that including only the top DEGs in a diagnostic model might lack a reliable distinguishing effect in all datasets, which has been one of the reasons transcriptome DEGs are currently difficult to use as MDD biomarkers.

Further in this study, four ML classifiers of screened feature genes were constructed. Among them, SVM produced better classification results. We then reduced the model size to 70 feature genes and found that the SVM model of these genes displayed acceptable performance in distinguishing MDD from control samples of discovery sets. The verification on an independent dataset exhibited an AUC of 0.78 and an accuracy of 0.67. The results showed that the predictive power of the model was superior to that of a single gene as an indicator of classification. The application of ML in some large-scale omics data is a popular undertaking, such as in cancer genomics (Zhou et al., 2018) and radiomics (Huang et al., 2018; Ding et al., 2019). In the depression-related studies, ML algorithms also have been used to find radiomics (Rubin-Falcone et al., 2018) and video- (Schultebraucks et al., 2020) and audio-based markers (Schultebraucks et al., 2020) for diagnosis or medication prediction. However, unlike cancers, in some studies of peripheral transcriptome biomarkers of MDD, it was difficult to find a relatively credible database such as TCGA (The Cancer Genome Atlas) as an independent validation. For example, one study constructed an elastic net model using immune-inflammatory signature to classify MDD and BD; it achieved high accuracy (AUC = 97%), but the result lacked the independent validation to acquire a true diagnostic effect of these biomarkers.

In contrast to other studies on MDD biomarkers, the blood transcriptomic data used for modeling here were from multiple studies, and the validation data were completely independent from the training data. Besides, we identified feature biomarkers by using meta-analysis followed by RF. Therefore, our strategy could help in identifying consistent and reliable biomarkers from different studies and was more conducive to the evaluation of the generalization ability of the model.

This study has several limitations. First, although existing data of MDD and healthy control subjects were used, it is unknown at this stage how much data are required to establish a reliable predictive model, which can be answered through empirical investigation. Second, the biomarkers used in this study were based on statistical significance, although the biological conception of these genes could also be considered. For example, our subsequent research can build a diagnostic model to refer to genes in the module of the PPI network. Third, because as many data as possible were used for modeling to improve the accuracy, independent validation data are needed in the future.

MDD occurs in a heterogeneous patient population, which makes accurate diagnosis a challenge. To address this challenge, we conducted meta-analysis of six datasets and found significant DEGs. The *TPST1*, *ARG1*, *KLRB1*, *WWC3*, *AKR1C3*, *MAF*, and *MAFG* genes were highlighted as potential feature genes influencing MDD. In addition, we constructed four ML models and chose SVM as a diagnostic model for MDD. We finally obtained an SVM diagnostic model containing 70 feature genes with an average AUC of 0.83, whose diagnostic effectiveness was superior to that of a single gene. Together, this study provided some markers that may be prospective precise diagnosis targets for MDD. Besides, this study provides new insight into how meta-analysis and ML can be used to find relatively objective transcriptional markers for complex mental diseases.

## Data Availability Statement

The datasets presented in this study can be found in online repositories. The names of the repository/repositories and accession number(s) can be found in the article/Supplementary Material.

## Author Contributions

SZ, ZB, XZ, and MX collected the data, conducted the analysis, and drafted the manuscript. ML provided resources and revised the manuscript. ZY administered project and revised the manuscript. All authors contributed to the article and approved the submitted version.

## Conflict of Interest

The authors declare that the research was conducted in the absence of any commercial or financial relationships that could be construed as a potential conflict of interest.

## Footnotes

1 https://www.ncbi.nlm.nih.gov/mesh

2 https://www.ncbi.nlm.nih.gov/gds/

3 http://www.bioconductor.org/

4 http://linus.nci.nih.gov/BRB-ArrayTools.html

5 http://www.reactome.org

6 http://www.cytoscape.org

7 http://cran.r-project.org/web/packages/caret

## Supplementary Material

Supplementary Figure 1 Quality control results of merged datasets from eight microarray profiles. SMR, standardized mean rank.

Supplementary Figure 2 Biological functional of protein–protein interaction network constructed using MDD-related DEGs. **(A)** Functional interaction (FI) network. Node sizes represent *p*-values of FI network, large for low value and small for high. Node colors define mean effect size in MDD-related DEGs, ranging from red for high expression to blue for low expression. **(B)** Five generated network modules incorporate 26 genes, which are shown in different colors in network modules. **(C)** Correlation heat map of genes in different modules.

Supplementary Figure 3 Workflow of machine learning. TP, true positive; TN, true negative; FP, false positive; FN, false negative.

Supplementary Figure 4 **(A)** The accuracy of gene selection procedure using RF. **(B)** Average AUC when modeling with different numbers of features. **(C)** ROC in model parameter calculation procedure using SVM.

Supplementary Figure 5 Clustering heatmap of feature genes and samples in training dataset. Color gradient from purple to red represents changes in expression from low to high. Bars represent samples (orange refers to MDD samples, blue to control samples).
