# Supplementary figures and images for: Identification of Diagnostic Markers for Major Depressive Disorder Using Machine Learning Methods
Source: Front Neurosci. 2021 Jun 18;15:645998. doi: 10.3389/fnins.2021.645998 (PMC8249859; doi:10.3389/fnins.2021.645998)

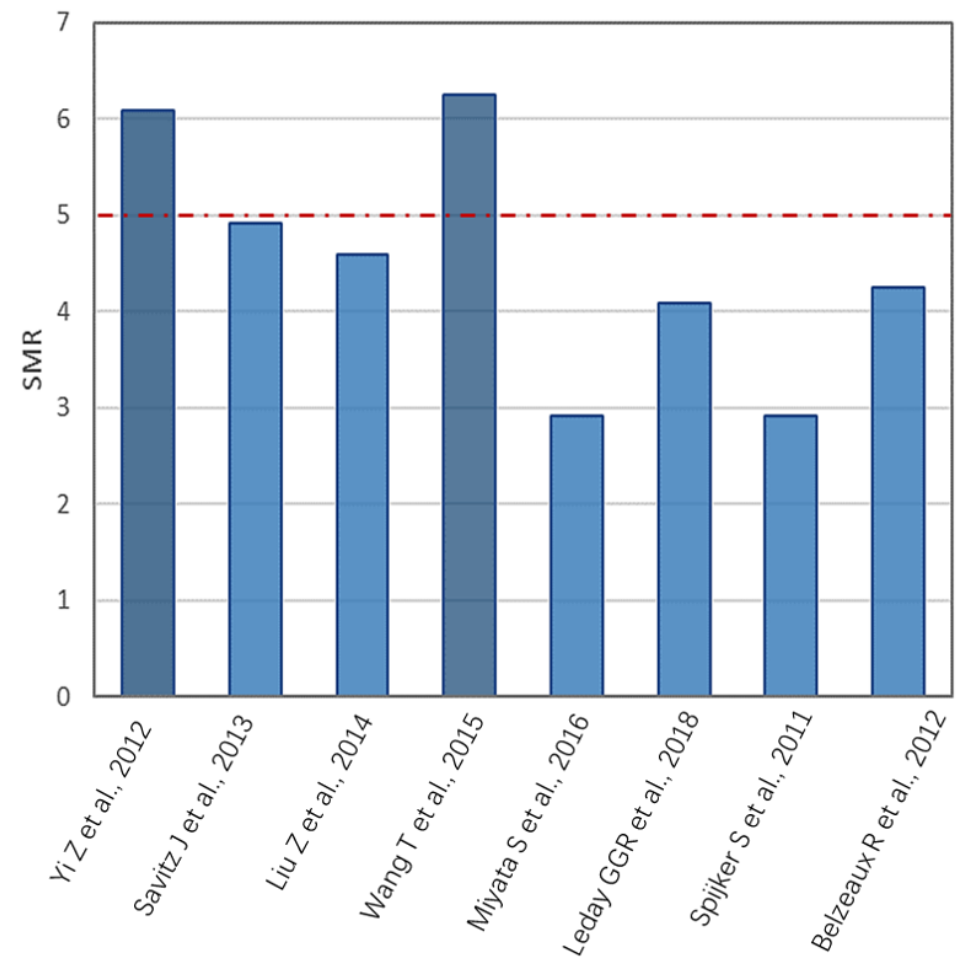

Figure S1

A

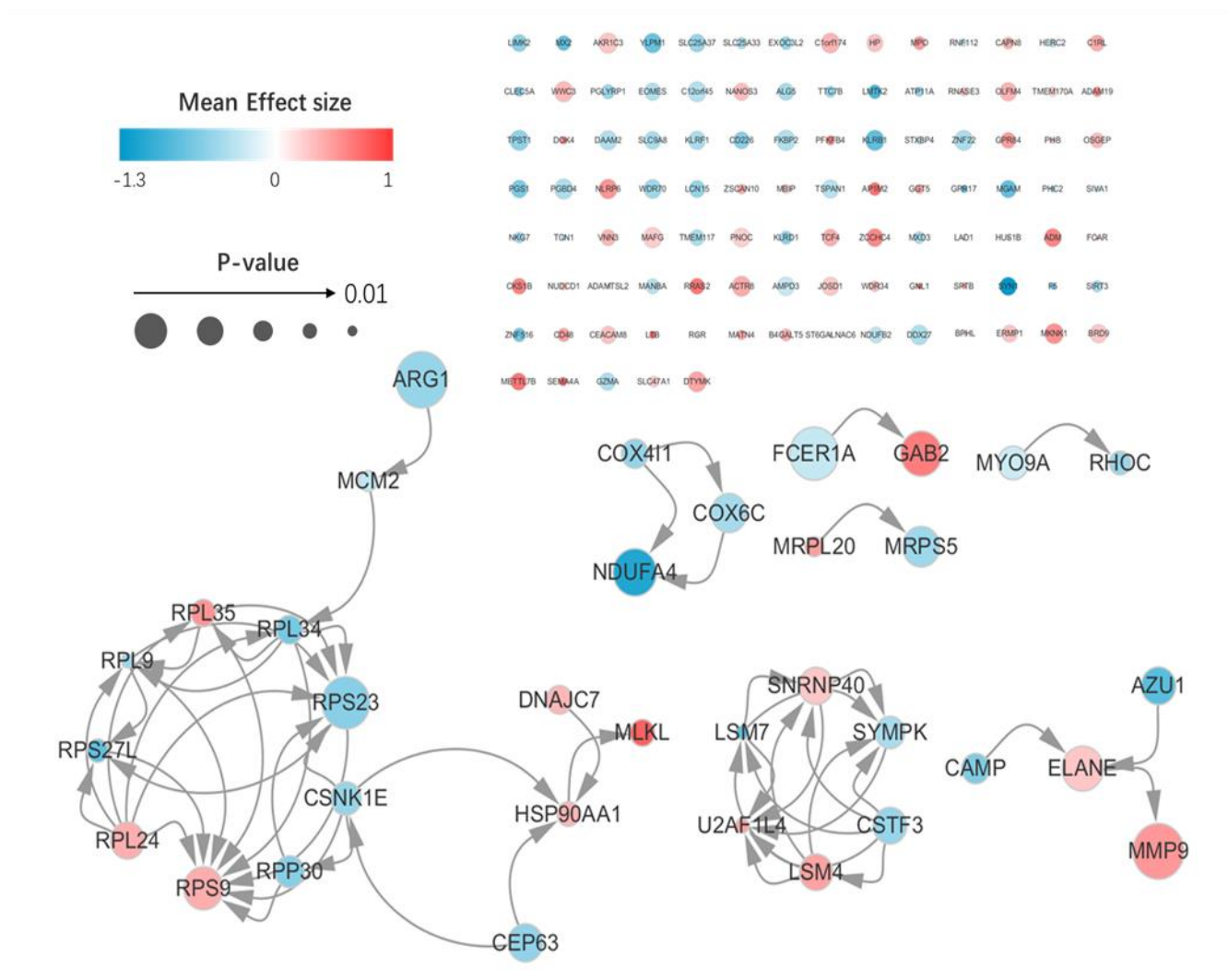

B

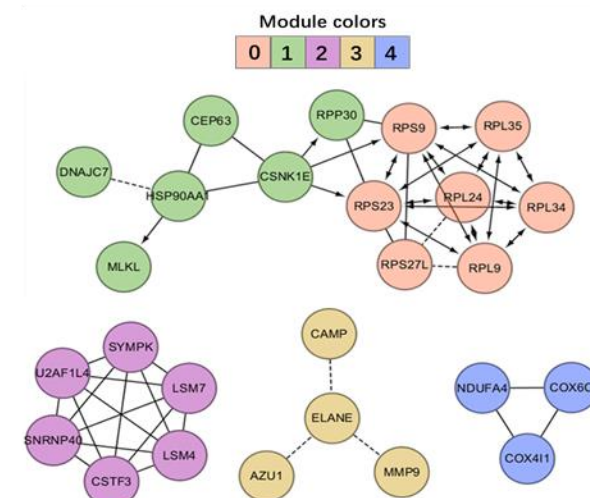

C

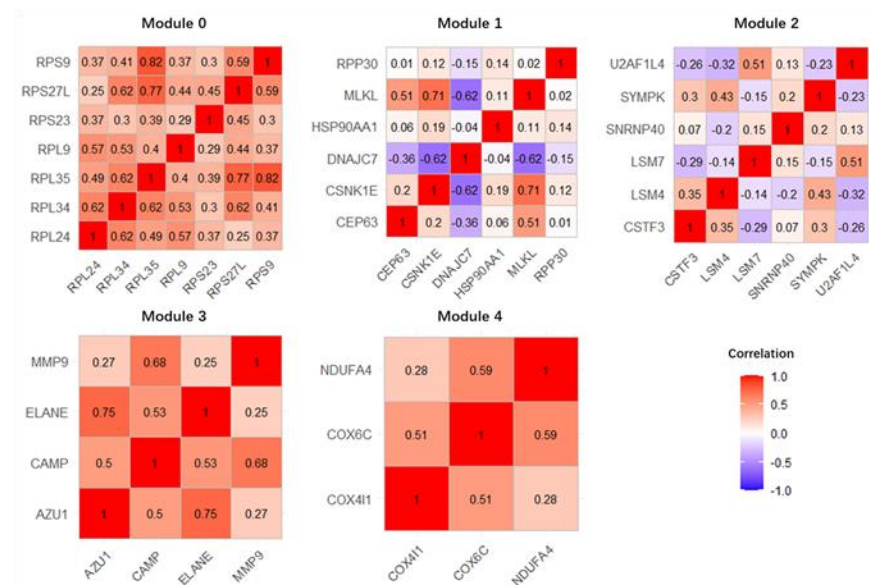

Figure S2

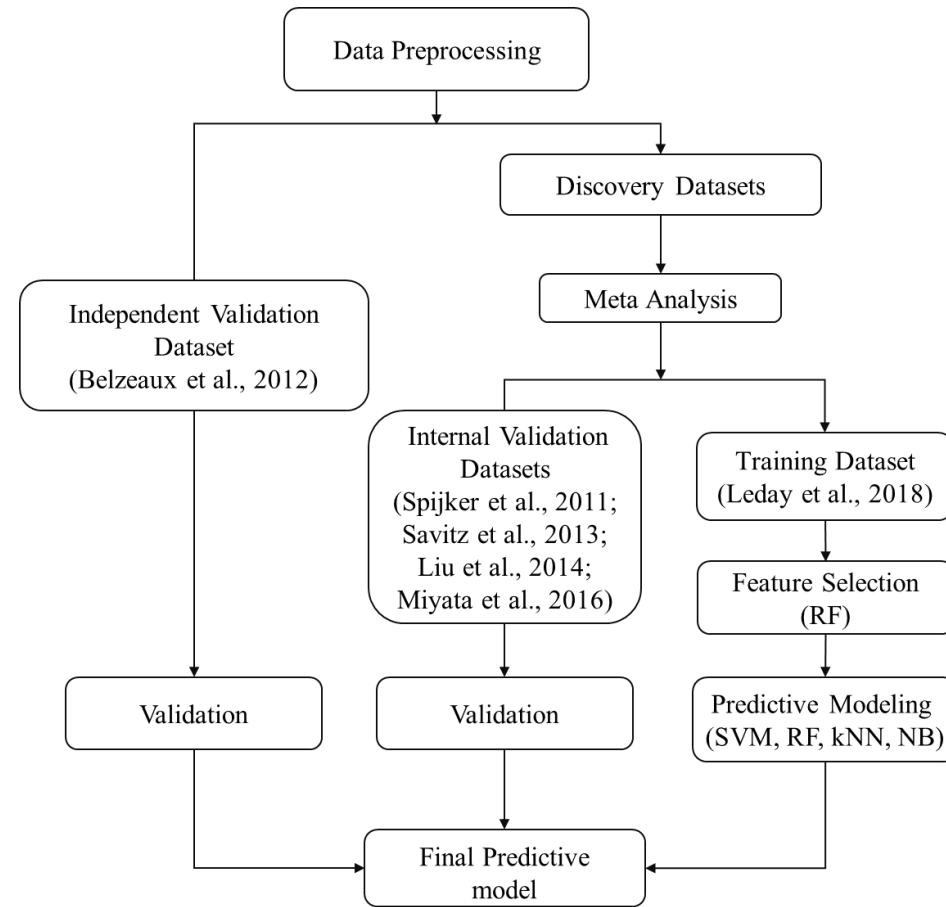

Figure S3

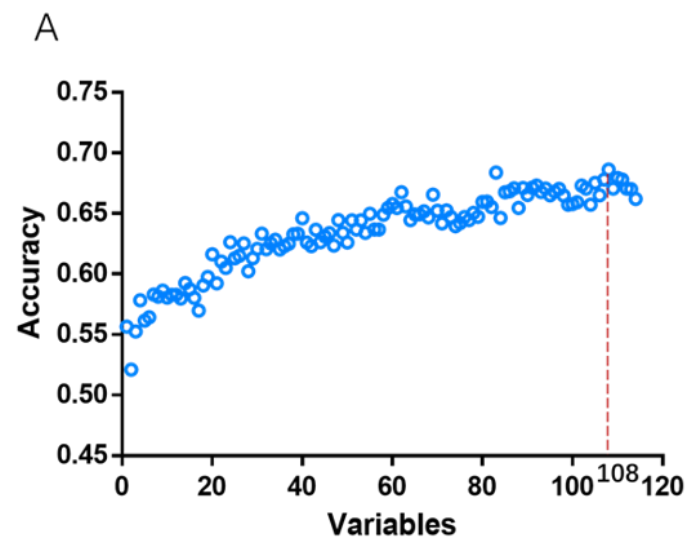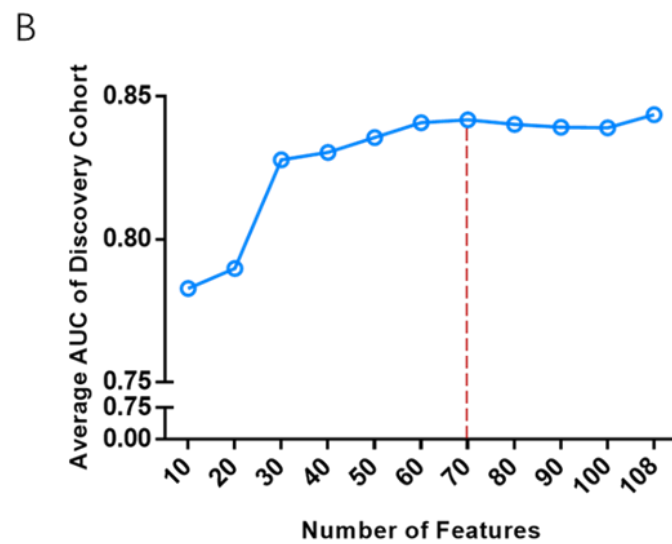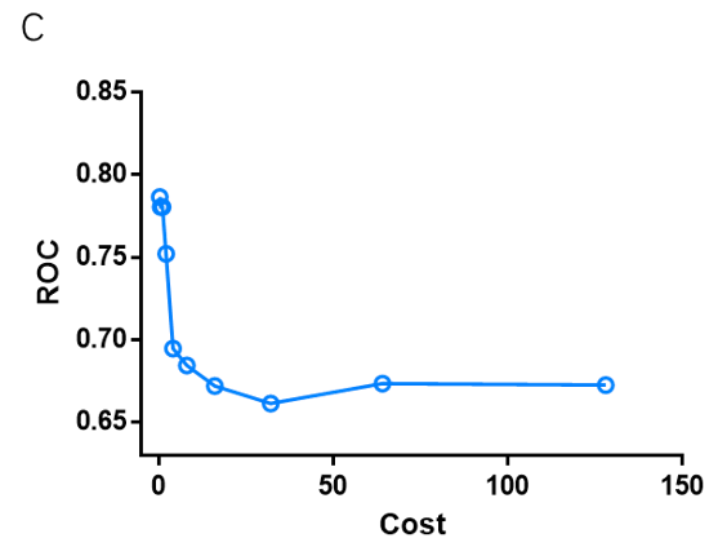

Figure S4

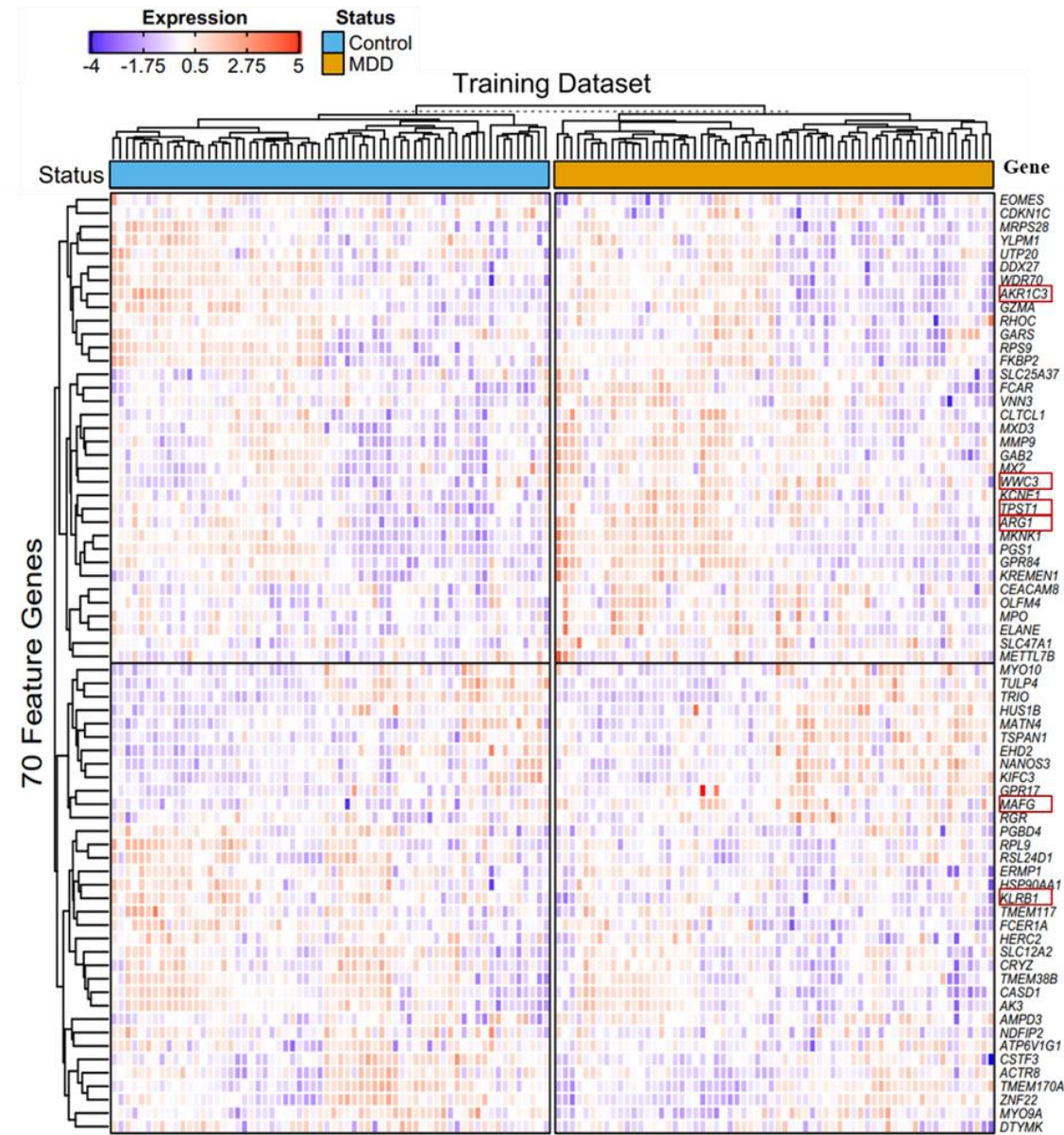

Figure S5

Supplement: Supplementary Figure 1 — Quality control results of merged datasets from eight microarray profiles. SMR, standardized mean rank. [file Image_1.pdf]
