# Supplementary material for: Identification of Diagnostic Markers for Major Depressive Disorder Using Machine Learning Methods
Source: Front Neurosci. 2021 Jun 18;15:645998. doi: 10.3389/fnins.2021.645998 (PMC8249859; doi:10.3389/fnins.2021.645998)
Supplement: Supplementary file 2 [file Data_Sheet_1.pdf]

Table S1. Results of quality control parameters and standardized mean rank

Study	IQC	EQC	AQCg	AQCp	CQCg	CQCp	SMR
Yi et al., 2012	2.4161	2.5830	0.6196	0.0077	0.0288	410	6.0833
Savitz et al., 2013	0.5252	0.2537	1.0470	1.7298	1.1844	410	4.9167
Liu et al., 2014	8.4785	8.8180	0.0244	1.1150	0.0529	410	4.5833
Wang et al., 2015	0.8947	0.7861	0.0579	1.1113	0.2141	410	6.2500
Miyata et al., 2016	4.1561	3.8496	1.3113	12.1410	2.2273	410	2.9167
Leday et al., 2018	2.8393	2.6361	1.0513	1.5851	0.9364	410	4.0833
Spijker et al., 2010	5.0325	5.6895	0.3120	14.5268	2.7962	410	2.9167
Belzeaux et al., 2012	7.1604	7.1453	0.0868	1.5600	0.1826	410	4.2500

QC = quality control; IQC = internal QC; EQC = external QC; AQCg = accuracy QC; AQCp = precision of AQCg; CQCg = consistency QC; CQCp = precision of CQCg; SMR = standardized mean rank.

Table S2. Differentially expressed genes after meta-analysis

Gene	Mean Effect Size	P-value	FDR	Regulation
TPST1	0.604007895	8.58E-07	0.0079493	up
ARG1	0.599582042	4.48E-06	0.0207697	up
KLRB1	-0.776312255	9.66E-06	0.0298172	down
WWC3	0.477393626	1.74E-05	0.0402783	up
MAFG	0.459591329	2.98E-05	0.0459433	up
AKR1C3	-0.528059765	2.53E-05	0.0459433	down
DTYMK	-0.604021136	9.03E-05	0.092888	down
ACTR8	-0.64725693	8.55E-05	0.092888	down
FKBP2	-0.797733323	7.49E-05	0.092888	down
MMP9	0.489424627	0.0001254	0.1102369	up
MKNK1	0.511166958	0.0001399	0.1102369	up
PGBD4	-0.52350488	0.0001428	0.1102369	down
C12orf45	-0.568185261	0.0002221	0.1469276	down
ZNF22	-0.522987459	0.0002177	0.1469276	down
C1orf174	-0.602154679	0.0002579	0.159278	down
YLPM1	-0.567015125	0.0003742	0.2166113	down
NLRP6	0.418479381	0.0004184	0.2225532	up
DDX27	-0.55344654	0.0004325	0.2225532	down
DAAM2	0.538840001	0.0006942	0.3215267	up
FCER1A	-0.296880745	0.0006802	0.3215267	down
RPS23	-0.584983182	0.0008672	0.365128	down
WDR70	-0.497738472	0.0008442	0.365128	down
OLFM4	0.38188839	0.0011075	0.3751992	up
AMPD3	0.454922725	0.0011197	0.3751992	up

SYN1	0.401829948	0.0012259	0.3751992	up
TSPAN1	0.318052537	0.0012602	0.3751992	up
ADM	0.66931151	0.0012791	0.3751992	up
SLC25A37	0.407517821	0.0014866	0.3751992	up
PGS1	0.562286237	0.0015089	0.3751992	up
SLC9A8	0.650382063	0.0015669	0.3751992	up
MGAM	0.721165303	0.0016505	0.3751992	up
CEACAM8	0.292743316	0.0017719	0.3751992	up
HP	0.300535434	0.0017865	0.3751992	up
NANOS3	0.370891822	0.0017933	0.3751992	up
METTL7B	0.354445164	0.0018392	0.3751992	up
LIMK2	0.784085429	0.001932	0.3751992	up
LCN15	0.439305863	0.0019404	0.3751992	up
TCF4	-0.603932671	0.0019848	0.3751992	down
ERMP1	-0.435924467	0.0019706	0.3751992	down
ALG5	-0.528604563	0.0019173	0.3751992	down
CD226	-0.276908461	0.0019073	0.3751992	down
NDUFA4	-0.795069157	0.00187	0.3751992	down
GZMA	-0.421851396	0.0017397	0.3751992	down
EOMES	-0.399750098	0.0017193	0.3751992	down
ZCCHC4	-0.528317496	0.0013501	0.3751992	down
JOSD1	-0.793048879	0.0012083	0.3751992	down
BRD9	-0.528374661	0.0011109	0.3751992	down
KLRF1	-0.431604694	0.0011038	0.3751992	down
PNOC	-0.544495001	0.0009964	0.3751992	down
GAB2	0.606260235	0.0023968	0.4202045	up
ELANE	0.27279156	0.0024194	0.4202045	up
VNN3	0.482453757	0.0024449	0.4202045	up

C1RL	0.668260622	0.0024612	0.4202045	up
GPR84	0.388507055	0.0025803	0.4202045	up
TMEM117	-0.396200932	0.0025857	0.4202045	down
RRAS2	-0.441462607	0.0025109	0.4202045	down
CKS1B	-0.50879298	0.0025104	0.4202045	down
NDUFB2	-0.413631977	0.0026962	0.4305982	down
RPS9	-0.462134985	0.0028525	0.4478424	down
MX2	0.415783835	0.003138	0.4825641	up
OSGEP	-0.602936836	0.0031778	0.4825641	down
MANBA	0.467212237	0.0032455	0.4848913	up
MRPS5	-0.683186134	0.0033836	0.4974946	down
PGLYRP1	0.401441333	0.00353	0.5109158	up
LMTK2	0.528726951	0.0037224	0.5135352	up
ZNF516	0.686575621	0.0038139	0.5135352	up
CEP63	0.4244462	0.0039037	0.5135352	up
AZU1	0.245647737	0.0039837	0.5135352	up
MPO	0.278475248	0.0041216	0.5135352	up
AP1M2	0.396357081	0.0041756	0.5135352	up
LSM4	-0.516195994	0.0042688	0.5135352	down
CSTF3	-0.304438254	0.0042244	0.5135352	down
KLRD1	-0.404174708	0.0041833	0.5135352	down
RPL24	-1.281323936	0.0041249	0.5135352	down
SNRNP40	-0.702521853	0.0041153	0.5135352	down
CD48	-0.424740495	0.0040376	0.5135352	down
COX6C	-0.442491069	0.0037153	0.5135352	down
EXOC3L2	0.340158511	0.0043456	0.5160675	up
B4GALT5	0.528261004	0.0048283	0.5492275	up
MXD3	0.318067883	0.0048712	0.5492275	up

CAPN8	0.335981558	0.0049018	0.5492275	up
CSNK1E	-0.796159077	0.0049213	0.5492275	down
MYO9A	-0.661838239	0.0048761	0.5492275	down
RPP30	-0.428963558	0.0052306	0.5569057	down
SIRT3	-0.542353588	0.0051856	0.5569057	down
SYMPK	-0.533968738	0.005179	0.5569057	down
SLC25A33	-0.368697017	0.0051425	0.5569057	down
TTC7B	0.296036305	0.0053814	0.5616427	up
SLC47A1	0.269920458	0.0055017	0.5616427	up
CAMP	0.355202464	0.0055579	0.5616427	up
ZSCAN10	0.329126859	0.0061028	0.5616427	up
ADAM19	0.413255975	0.0061112	0.5616427	up
MATN4	0.274111966	0.0061325	0.5616427	up
PFKFB4	0.325683082	0.0062884	0.5616427	up
MLKL	0.405900717	0.0062946	0.5616427	up
GGT5	0.543278997	0.0063427	0.5616427	up
CLEC5A	0.231231457	0.0063588	0.5616427	up
ATP11A	0.615341778	0.0063665	0.5616427	up
TMEM170A	-0.384637318	0.0062958	0.5616427	down
RPL35	-1.110738197	0.006262	0.5616427	down
DNAJC7	-0.523443758	0.0059525	0.5616427	down
RPL34	-0.913149588	0.005931	0.5616427	down
COX4I1	-0.82129961	0.0057866	0.5616427	down
NKG7	-0.40328595	0.005685	0.5616427	down
WDR34	-0.381076949	0.0055458	0.5616427	down
MBIP	-0.825511698	0.0065006	0.5680648	down
SEMA4A	0.275824442	0.0067236	0.5820589	up
HSP90AA1	-0.259873457	0.006793	0.5826298	down

RNASE3	0.301871004	0.0070216	0.5912791	up
RHOC	-0.350111347	0.0070165	0.5912791	down
RPS27L	-0.554979498	0.007109	0.593248	down
HERC2	-0.295319476	0.0071907	0.5947085	down
DOK4	0.587664701	0.0075365	0.6007185	up
GPR17	0.240178175	0.0075935	0.6007185	up
MCM2	-0.46288989	0.0076525	0.6007185	down
NUDCD1	-0.383775484	0.0076049	0.6007185	down
LTB	-0.460046237	0.00753	0.6007185	down
STXBP4	-0.315463865	0.0073421	0.6007185	down
MRPL20	-0.467283968	0.0078061	0.6076268	down
RNF112	0.645641704	0.008124	0.6271032	up
PHC2	0.3653527	0.0082658	0.6280032	up
F5	0.704482286	0.0082712	0.6280032	up
GNL1	-0.460124427	0.0085548	0.639059	down
PHB	-0.819984412	0.0085341	0.639059	down
SPTB	0.23639201	0.0089348	0.6453341	up
TCN1	0.258902271	0.009112	0.6453341	up
BPHL	-0.428350482	0.0091265	0.6453341	down
RPL9	-0.545473187	0.0090778	0.6453341	down
LSM7	-0.760823743	0.0090225	0.6453341	down
U2AF1L4	-0.492637911	0.0089369	0.6453341	down
SIVA1	-0.565724372	0.0087913	0.6453341	down
ADAMTSL2	0.200372511	0.0096025	0.6718348	up
LAD1	0.454544371	0.0097673	0.6718348	up
HUS1B	0.295416932	0.0098214	0.6718348	up
FCAR	0.57371573	0.0099186	0.6718348	up
RGR	0.322760098	0.0099365	0.6718348	up

ST6GALNAC6	-0.461274021	0.0098851	0.6718348	down

Table S3. Basic information of six most significant DEGs

Gene	Gene name	Molecular Function	Biological Process	Related psychiatric disorders
TPST1	Tyrosylprotein sulfotransferase 1	sulfotransferase activity; transferase activity; protein homodimerization activity	peptidyl-tyrosine sulfation; inflammatory response; 3'-phosphoadenosine 5'-phosphosulfate metabolic process	
ARG1	Arginase 1	arginase activity; hydrolase activity;	urea cycle; arginine metabolic process; immune system process; response to steroid hormone	
KLRB1	Killer cell lectin like receptor B1	transmembrane signaling receptor activity; carbohydrate binding; protein binding	regulation of immune response; cell surface receptor signaling pathway	
WWC3	WWC family member 3	kinase binding; molecular adaptor activity	negative regulation of transcription by RNA polymerase II; negative regulation of hippo signaling; negative regulation of organ growth	
AKR1C3	Aldo-keto reductase family 1 member C3	aldo-keto reductase (NADP) activity; oxidoreductase activity; interconvert active on androgens, estrogens and progestins with their cognate inactive metabolites	steroid metabolic process; prostaglandin metabolic process; progesterone metabolic process; G protein-coupled receptor signaling pathway	Bipolar disorders
MAFG	MAF bZIP transcription factor G	DNA-binding transcription factor activity	regulation of transcription, DNA-templated; regulation of cell proliferation	

Table S4. KEGG pathways enriched in the modules

Module	Genes included in module	KEGG term	FDR
0	*RPL24*, *RPL34*, *RPL35*, *RPL9*, *RPS23*, *RPS27L*, *RPS9*	Ribosome	6.937E-13
1	*CEP63, CSNK1E, DNAJC7, HSP90AA1, MLKL, RPP30*	Circadian rhythm	2.920E-02
		Antigen processing and presentation	3.910E-02
		IL-17 signaling pathway	4.700E-02
		Progesterone-mediated oocyte maturation	4.950E-02
2	*CSTF3, LSM4, LSM7, SNRNP40, SYMPK, U2AF1L4*	Spliceosome	1.893E-06
		RNA degradation	8.795E-04
		mRNA surveillance pathway	8.795E-04
3	*AZU1, CAMP, ELANE, MMP9*	Transcriptional misregulation in cancer	1.470E-02
4	*COX4I1, COX6C, NDUFA4*	Oxidative phosphorylation	4.843E-06
		Parkinson disease	4.843E-06
		Cardiac muscle contraction	1.596E-04
		Alzheimer disease	4.843E-06
		Huntington disease	4.843E-06
		Metabolic pathways	2.029E-03

Table S5. DEGs after meta-analysis of discovery sets

Gene	Mean Effect Size	P-value	Regulation
TPST1	0.659128477	9.42E-07	up
KCNE1	0.568851661	4.00E-06	up
ARG1	0.571137998	1.37E-05	up
AKR1C3	-0.600148572	2.03E-05	down
WWC3	0.481138123	2.81E-05	up
MAFG	0.459700392	4.83E-05	up
HLA-DRA	-0.609672652	7.87E-05	down
MMP9	0.550162751	0.000138709	up
WDR61	-0.492153493	0.000147039	down
FCER1A	-0.41825094	0.000241801	down
CRYZ	-0.564139531	0.000278604	down
RSL24D1	-0.664229542	0.000299342	down
ZNF22	-0.545609603	0.000322771	down
CLTCL1	0.470160318	0.00041185	up
C12orf45	-0.565468347	0.000458557	down
NLRP6	0.449187389	0.000515458	up
KLRB1	-0.716922316	0.000528859	down
DTYMK	-0.473608443	0.000554668	down
ZCCHC4	-0.622063759	0.000567788	down
PGBD4	-0.530156442	0.000597225	down
MKNK1	0.377705844	0.000752494	up
SYN1	0.46500882	0.001003068	up
C1orf174	-0.512190614	0.001124874	down
FKBP2	-0.795480505	0.001213034	down
CD226	-0.335151592	0.001320404	down
PNOC	-0.579627232	0.001379222	down

YLPM1	-0.596204372	0.001403839	down
TSPAN1	0.334519658	0.001450642	up
DDX27	-0.482305947	0.001491573	down
CSTF3	-0.417206066	0.001573906	down
GARS	-0.4905895	0.001591884	down
NDUFB2	-0.512616235	0.001719823	down
HAVCR2	-0.497288686	0.00175138	down
TRIO	-0.538581448	0.001821686	down
HP	0.301173783	0.002188307	up
SLC25A37	0.407527411	0.002205936	up
RPL9	-0.669885916	0.002208214	down
WDR70	-0.446945373	0.002226832	down
EOMES	-0.409319363	0.002317447	down
KLRF1	-0.38738848	0.002470583	down
SLC12A2	-0.525341457	0.002584875	down
TMEM38B	-0.446242841	0.002596752	down
MATN4	0.384575111	0.002628873	up
SRFBP1	-0.484002861	0.002932469	down
ACTR8	-0.632563605	0.002935339	down
METTL7B	0.359578461	0.003032678	up
LCN15	0.469148091	0.00306696	up
NANOS3	0.351288863	0.003068937	up
CEACAM8	0.255690008	0.003122831	up
EXOC3L2	0.403815387	0.003378161	up
AMPD3	0.369908883	0.003496633	up
AK3	-0.58411055	0.003610117	down
OLFM4	0.270448811	0.003700983	up
UTP20	-0.467144453	0.003843181	down

RPS9	-0.483326267	0.003882648	down
ZNF572	-0.364939485	0.003948109	down
MYO9A	-0.508434668	0.003994752	down
AZU1	0.260057411	0.003998933	up
RHOC	-0.455005743	0.004030772	down
CASD1	-0.400003826	0.004041058	down
PRIM2	-0.522411923	0.00405113	down
GPR17	0.311544752	0.004160926	up
ERMP1	-0.395730894	0.004219201	down
ELANE	0.222443307	0.004321655	up
SLC47A1	0.316204685	0.004639751	up
ATP6V1G1	-0.516630273	0.004680166	down
HLA-DMB	-0.367112842	0.004802003	down
SLC25A33	-0.414932354	0.004911607	down
CHML	-0.345917607	0.005105417	down
MXD3	0.342552183	0.00511572	up
GPR84	0.339933816	0.005421292	up
LSM4	-0.58778104	0.005492968	down
TMEM117	-0.361934066	0.005513238	down
DAAM2	0.537803125	0.005617068	up
VNN3	0.440726067	0.005617849	up
PGS1	0.606677008	0.005703877	up
FCAR	0.421122167	0.005961099	up
HSP90AA1	-0.295277679	0.005988095	down
GZMA	-0.3062144	0.006128057	down
HLA-DPA1	-0.460286215	0.006147061	down
PHLDA3	0.327513241	0.006355774	up
RPS23	-0.6142547	0.006376791	down

TULP4	-0.461835931	0.006449599	down
ALG5	-0.407699527	0.006701179	down
EHD2	0.389773157	0.006715111	up
CKS1B	-0.449228043	0.006755117	down
GAB2	0.501942557	0.007158824	up
HERC2	-0.321996291	0.007168411	down
SCRN1	-0.610125291	0.00721768	down
TMEM170A	-0.411455762	0.007269933	down
CLEC5A	0.283463325	0.007323382	up
HUS1B	0.343114607	0.00743933	up
MRPS28	-0.25672525	0.00746465	down
NDFIP2	-0.351476607	0.007628619	down
TRIAP1	-0.409282854	0.00785918	down
CDKN1C	-0.339903574	0.007885642	down
NKG7	-0.406023382	0.008154152	down
RGR	0.384141298	0.008161116	up
TMEM192	-0.457516795	0.008319474	down
COX6C	-0.39150565	0.008434569	down
AGK	-0.668787738	0.008651241	down
MPO	0.204871478	0.008688306	up
MYO10	0.323034436	0.008943254	up
NUDCD1	-0.459411255	0.00896014	down
MX2	0.324199199	0.009063755	up
KIFC3	0.352045843	0.009083814	up
NUP37	-0.459486827	0.009218487	down
GH1	0.500490027	0.009372817	up
N4BP2L2	0.533531886	0.009436419	up
CEP63	0.357536728	0.009596737	up

AP1S2	-0.387126947	0.009653541	down
NUDT9	-0.392985202	0.009735896	down
KREMEN1	0.509268847	0.009930603	up
ADM	0.599175229	0.009990152	up

Table S6. A list of hyperparameters used in the study on ML

Model	Hyperparameter	Search Space	Optimal or Default
SVM	C: Cost	[0.25, 0.50, 1.00, 2.00, 4.00, 8.00, 16.00, 32.00, 64.00, 128.00]	0.25
	sigma: Kernel coefficient for 'poly'		0.00910
RF	mtry: Number of variables randomly sampled as candidates at each split	[2, 13, 25, 37, 49, 60, 72, 84, 96, 108]	2
	ntrees: Number of trees to grow		500
	nodesize: Minimum size of terminal nodes		1
	maxnodes: Maximum number of terminal nodes trees in the forest can have.		Null
kNN	kmax: Maximum number of k	[5, 7, 9, 11, 13, 15, 17, 19, 21, 23]	23
	distance: Parameter of Minkowski distance		2 (Euclidean Distance)
	kernel: Kernel to use (weighted algorithm)		“optimal”
NB	fL: Factor for Laplace correction		0
	usekernel: Weather a kernel density estimate is used for density estimation	[FALSE, TRUE]	TRUE
	adjust: The bandwidth of the kernel density		1

Table S7. Expression of 70 feature genes in training dataset

Gene	Fold Change	P-value	Mean Decrease Accuracy
MAFG	1.206329721	2.18251E-05	0.003658186
KCNE1	1.378509561	3.25535E-05	0.006102351
KLRB1	0.795495385	4.20243E-05	0.004082334
TPST1	1.31970914	0.000232921	0.001521426
TSPAN1	1.115686845	0.001041665	0.00308362
ARG1	1.590427523	0.00106927	0.001123077
GPR17	1.133115651	0.001759195	0.004931524
HSP90AA1	0.87510687	0.001932004	0.002049337
MMP9	1.358608934	0.002312538	0.000960697
RPL9	0.835605987	0.002717355	0.001135081
PGBD4	0.858177746	0.002848935	0.000973958
CLTCL1	1.203266733	0.003323706	0.000463788
WWC3	1.110760147	0.003417174	0.000570701
NDFIP2	0.856129092	0.003472665	0.000647549
MPO	1.27325805	0.003989524	0.001707852
FCER1A	0.760956608	0.004644569	0.001006669
EOMES	0.859565996	0.004876531	0.00063949
FCAR	1.312453718	0.006963714	0.005736951
CSTF3	0.884509413	0.007422764	0.000641987
MXD3	1.106599163	0.008324053	0.000775779
CEACAM8	1.560209323	0.00841224	0.001206283
NANOS3	1.1146328	0.008800296	0.000594798
RSL24D1	0.779997283	0.009236312	0.002455201
ELANE	1.22002393	0.009313236	0.00035262

MKNK1	1.16641815	0.009915303	0.001004088
GAB2	1.143692274	0.010006203	0.000500317
KREMEN1	1.257288006	0.011114681	0.000704504
MX2	1.099591806	0.011213854	0.001354795
SLC47A1	1.231633067	0.011637111	0.002540974
ERMP1	0.871340729	0.011827449	0.001098482
MYO9A	0.86326041	0.01346018	0.000898795
CASD1	0.738426941	0.01349044	0.000726276
OLFM4	1.695789587	0.015207858	0.000465444
GZMA	0.802310238	0.015484672	0.000655546
UTP20	0.906573741	0.016178396	0.000444135
TMEM117	0.888845372	0.016545125	0.001013144
SLC25A37	1.159461824	0.019625163	0.000814034
MRPS28	0.858173255	0.020245154	0.001207249
METTL7B	1.125365456	0.021345057	0.00059253
GPR84	1.248017015	0.024256544	0.00321704
AKR1C3	0.761188198	0.02549659	0.000940512
ZNF22	0.863400908	0.025994284	0.000391257
RGR	1.087435487	0.027921428	0.000408086
CRYZ	0.853775936	0.028204469	0.000729331
HERC2	0.899705147	0.029574879	0.000320129
TMEM38B	0.827599657	0.033236687	0.001668221
ACTR8	0.869351353	0.036890159	0.000700642
DTYMK	0.868050486	0.037984905	0.000484728
KIFC3	1.100630119	0.038510645	0.00050595
AMPD3	1.084898264	0.043342421	0.001009457
TMEM170A	0.920666239	0.04870703	0.000697008
MATN4	1.077103762	0.049979905	0.001323169

AK3	0.872101757	0.056231114	0.000550907
WDR70	0.920111597	0.067444355	0.000429048
HUS1B	1.090748128	0.072150434	0.000968124
DDX27	0.928949627	0.083122204	0.000458874
YLPM1	0.905720882	0.091505707	0.001074808
MYO10	1.139577725	0.111639789	0.000565801
GARS	0.948423908	0.11246073	0.002383729
EHD2	1.106422465	0.119743862	0.000820297
VNN3	1.116918708	0.192913503	0.001904376
PGS1	1.097505937	0.251937963	0.001011834
SLC12A2	0.926596829	0.285011883	0.000405274
TULP4	0.944450626	0.330295258	0.000463054
CDKN1C	0.928414744	0.346102895	0.000309616
TRIO	0.94000321	0.416894757	0.00037473
RPS9	0.971576794	0.423659864	0.000778739
RHOC	0.95558217	0.482201349	0.000288408
FKBP2	0.969826531	0.506739673	0.000719759
ATP6V1G1	0.960869626	0.547555352	0.000497441

Table S8. Positive predictive value (PPV) and Matthews correlation coefficient (MCC) of the SVM model

Testing Samples	Study	PPV	MCC
Training	Leday et al., 2018	0.77	0.55
Internal test	Spijker et al., 2010	0.65	0.35
	Savitz et al., 2013	0.73	0.62
	Liu et al., 2014	0.82	0.73
	Miyata et al., 2016	0.78	0.54
Independent test	Belzeaux et al., 2012	0.67	0.33
Mean ± SD		0.74 ± 0.06	0.52 ± 0.14

Table S9. The classification ability of randomly selected 70 genes

Random Times	AUC	Accuracy	Sensitivity	Specificity
1	0.59	0.56	0.67	0.44
2	0.65	0.67	0.78	0.56
3	0.57	0.44	0.44	0.44
Mean	0.61	0.56	0.63	0.48
